# The Role of the Ecto-Nucleotidases CD73 and CD39 in Chemo- and Immunotherapy

**DOI:** 10.3390/cancers18060957

**Published:** 2026-03-16

**Authors:** Patryk T. Mucha, Ankita Brahmachari, Marika A. Frańczak, Marta Tomczyk, Barbara Kutryb-Zając, Patrycja Koszałka, Elisa Giovannetti, Godefridus J. Peters

**Affiliations:** 1 Department of Biochemistry, Medical University of Gdansk, 80-210 Gdansk, Poland; 2 Department of Medical Oncology, Amsterdam UMC, Vrije Universiteit Amsterdam, 1081 HV Amsterdam, The Netherlands; 3 Liberal Arts and Sciences, Amsterdam University College, 1098 XG Amsterdam, The Netherlands; 4 Centre of Experimental Cardiooncology, Medical University of Gdansk, 80-210 Gdansk, Poland; 5 Department of Cell Biology and Immunology, Medical University of Gdansk, 80-210 Gdansk, Poland; pkosz@gumed.edu.pl; 6 Cancer Pharmacology Lab, Fondazione Pisana per La Scienza, 56017 Pisa, Italy

**Keywords:** immunotherapy, chemotherapy, CD73, CD39, adenosine, non-small cell lung cancer

## Abstract

Immunotherapy, particularly effective in tumors with a high mutational burden, is very often administered in combination with chemotherapy. Several tumor types with a high mutational rate include melanoma and non-small cell lung cancer (NSCLC), which are particularly sensitive to immunotherapy. For NSCLC, conventional platinum-based doublet chemotherapy has been extended with drugs targeting signaling pathways (such as the epidermal growth factor receptor) and immune checkpoint inhibitors (ICI) directed against PD-1 and PD-L1. This review highlights the potential role of the membrane antigens CD73 and CD39 in enhancing the efficacy of combined immuno-chemotherapy. These ecto-nucleotidases catalyze the degradation of extracellular ATP to AMP and subsequently to adenosine (Ado), a potent immunosuppressive metabolite that acts through adenosine receptors. Consequently, CD73 and CD39 function as key downregulators of immunogenic signaling. Both CD73 and CD39 are highly expressed not only on tumor cells but also on immune and endothelial cells within the tumor microenvironment. Conventional chemotherapy may further upregulate their expression, contributing to drug resistance and impaired immune responses. To counteract these effects, inhibitors of CD73 and CD39, both monoclonal antibodies and small molecules, are currently under clinical evaluation, with early results indicating potential therapeutic benefit. Although this evidence supports the involvement of CD73 and CD39 in modulating responses to immunotherapy, particularly in combination with chemotherapy, the precise mechanisms underlying these interactions remain unclear. Elucidating these pathways will be critical for optimizing treatment strategies and improving clinical outcomes in malignancies such as NSCLC. This review highlights the critical role of these pathways in optimizing treatment strategies and improving clinical outcomes in malignancies such as NSCLC.

## Simple Summary

Immunotherapy has become an important treatment option for cancers with many genetic changes, such as melanoma and non-small cell lung cancer, and is often combined with chemotherapy. While these treatments can be effective, not all patients respond equally well. This review focuses on two proteins, CD73 and CD39, that are found on cancer cells and surrounding cells in the tumor environment. These proteins produce adenosine, a substance that weakens the immune response against tumors and may reduce the efficacy of cancer therapies. Chemotherapy can further increase the levels of these proteins, potentially contributing to treatment resistance. New drugs that block CD73 and CD39 are currently being tested and show promising early results. Understanding how these proteins influence combined cancer treatments may help improve future therapies and lead to better outcomes for patients.

## 1. Introduction

### 1.1. Biology of the CD39/CD73-Adenosine Axis in Relation to Immune Regulation

**Figure 1:**
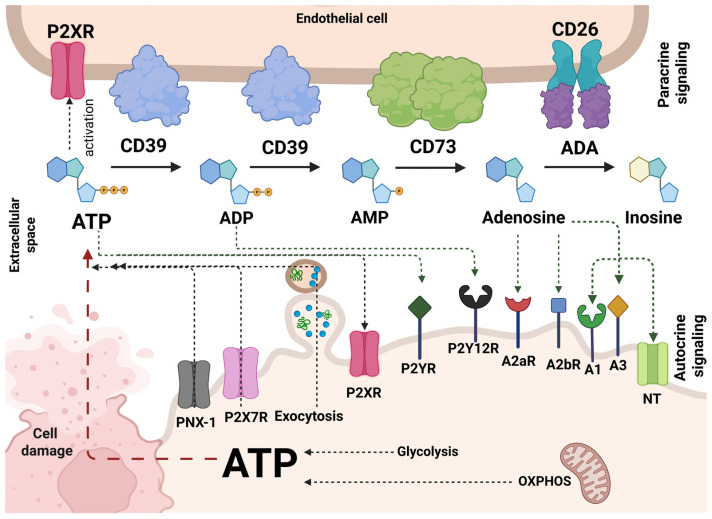
Inside cells, oxidative phosphorylation (OXPHOS) and glycolysis drive ATP production. Various pathways, including but not limited to exocytosis, cell damage, PNX-1 hemichannels, and P2X7R, facilitate the ATP release into the extracellular space. Extracellular ATP then activates ligand-activated ion channels (P2XR) and P2YR receptors. CD39 and CD73 sequentially degrade ATP to ADP and AMP and then to adenosine (ADO). P2Y12R can be activated by ADP, and ADO can activate G-protein-coupled receptors (GPCRs) in the P1 family (A2aR, A2bR). ADO is broken down to inosine by adenosine deaminase (ADA), which can be anchored to the cell surface via interaction with CD26 (also known as dipeptidyl peptidase IV, DPPIV), while nucleoside transporters (NT) enable an uptake back into the cell [2,3]. Created with BioRender.com.

A key metabolic pathway in tumor immunity regulation is the signaling of extracellular adenosine and its extra- and intracellular metabolism (Figure 1). Adenosine is a product of the extracellular membrane-bound nucleotidases CD39 and CD73. The ecto-nucleotidase CD39 degrades the pro-inflammatory adenosine triphosphate (ATP) to adenosine monophosphate (AMP), which is subsequently broken down to adenosine by the ecto-5′-nucleotidase CD73 [1]. Adenosine is a signaling molecule acting through P1 purinergic receptors: A1, A2a, A2b, and A3 (Figure 1).

As a rate-limiting enzyme in the ATP-to-adenosine conversion cascade, CD73 is upregulated under hypoxia, inflammation, mitogenic signals, EMT, and BRAF-driven oncogenesis [4,5] resulting in an enhanced accumulation of extracellular adenosine. Adenosine itself suppresses the immune system through interaction with the A2a (A2aR) and the A2b receptor (A2bR) [1], and also through its interaction with the adenosine receptors A1 and A3, while adenosine itself may have effects on specific subsets of immune cells. In melanoma, activation of the AdoA2aR has been associated with immunosuppressive effects through an increase in cAMP levels, whereas stimulation of the AdoA3R has been shown to enhance the antitumor activity of NK and T cells [6]. Receptors A1, A2a, and A3 can be activated under physiological adenosine concentrations; however, activation of the low-affinity A2b receptor can be achieved only at high, pathological concentrations. Adenosine is also an excellent substrate for adenosine deaminase (ADA), known to play an important role in immune function. Extracellular ADA can be anchored to the cell surface through interaction with CD26 (dipeptidyl peptidase IV, DPPIV), which positions the enzyme to efficiently metabolize adenosine in the pericellular space [3].

Cellular uptake of extracellular adenosine can be mediated by various transporters, such as facilitated diffusion catalyzed by the equilibrative nucleoside transporter (ENT), while the energy-dependent concentrative transporters also play a role in the uptake [7]. Earlier, it was demonstrated that intracellular adenosine (and its deoxy form, deoxyadenosine) can affect cellular functions of B- and T cells. Accumulation of intracellular adenosine and deoxyadenosine can affect the immune system, especially under conditions of a deficiency of adenosine deaminase (ADA), which degrades adenosine to inosine, or of purine nucleoside phosphorylase (PNP), which degrades inosine to hypoxanthine. A deficiency of ADA has been associated with severe combined immunodeficiency (SCID), a severe dysfunction of T and B cells, while a deficiency of PNP is associated with severe T-cell dysfunction [8,9]. This immune deficiency has been linked to an imbalance of both pyrimidine and purine nucleotide pools [10,11], of which the increase in dATP seems to be the most predominant factor in killing thymocytes in their early development to T-cells [10]. So, low or high ADA/PNP activity may affect the function of CD73 by either no or rapid degradation of adenosine. The way adenosine is transported and broken down may affect the adenosinergic pathway and determine the function of adenosine in the immune system.

### 1.2. Current Treatment Paradigms in Chemo- and Immunotherapy of NSCLC

Immunotherapy is particularly active against tumors with a high mutational burden, such as lung cancer and melanoma [12,13]. For melanomas, the use of CTLA-4-directed therapies with ipilimumab was a breakthrough [14], as well as the BRAF V600E-directed tyrosine kinase inhibitor (TKI), vemurafenib, and the MEK inhibitor, trametinib [15]. More recent studies showed even higher efficacy with the programmed cell death protein 1 (PD-1)-directed antibodies nivolumab and pembrolizumab [16,17], both in adjuvant and neo-adjuvant therapies, alone and in combination [18]. These therapies have replaced earlier therapies using chemotherapy and interferon-alpha.

However, for lung cancer, the picture is different. Lung cancer remains one of the leading causes of cancer-related deaths worldwide [19], with about 92,000 deaths in 2022 in the USA [20]. There are two main forms of primary lung cancer: non-small cell lung cancer (NSCLC), accounting for about 87%, and small-cell lung cancer (SCLC), which are classified based on the type of cancer cells [21]. NSCLC includes squamous carcinomas, adenocarcinomas (also called non-squamous), and large-cell carcinomas [22]. Unfortunately, most NSCLC cases are diagnosed at advanced stages, and these patients have a poor prognosis with an average overall survival time of less than 3 years [23].

The most widely used treatment options for NSCLC include surgery, radiation, conventional chemotherapy, targeted therapy, and, most recently, immunotherapy; other therapies are still under investigation [22,24,25]. Treatment with conventional chemotherapy, usually platinum-based doublets, has historically shown a response rate of 29% [26]. These doublets usually consist of cisplatin or carboplatin in combination with a taxane (paclitaxel or docetaxel), gemcitabine, vinorelbine, or irinotecan, but, for non-squamous NSCLC and mesothelioma, pemetrexed is preferred [27]. However, despite initially being effective, conventional chemotherapy has a limited effect on the overall survival of NSCLC patients, with a 2-year overall survival of 31%. Targeted therapy, usually with a tyrosine kinase inhibitor (TKI), is effective against subsets of adenocarcinomas, such as patients with activating mutations in the epidermal growth factor receptor (EGFR) and no K-Ras mutations. EGFR-TKI therapy with erlotinib or gefitinib was used as first-line treatment for patients with this mutation [28]. Regardless of the initial response, acquired resistance after 9–18 months to erlotinib or gefitinib seems to be unavoidable and is usually caused by a T790M mutation. As a result, the development of innovative therapeutic approaches is directed to the T790M mutation or targeting alternative pathways. Current guidelines actually advise giving the 3rd generation EGFR-TKI, osimertinib, which is targeted against this mutation [29,30]. Patients with an EML4-ALK fusion gene (up to 5% of NSCLC patients) were previously treated with crizotinib, but resistance to crizotinib occurs after approximately 8 months. However, patients are usually sensitive to a next-generation inhibitor, which is directed to specific mutations and includes ceritinib, lorlatinib, or brigatinib [31].

Patients with a K-Ras mutation are not eligible for an EGFR-TKI and will receive a platinum doublet [29,31], although specific K-Ras mutant inhibitors are now also available [32]. Targeted therapy has shown a large benefit for subgroups of patients, but unfortunately, this does not hold for a large group of patients without targetable mutations. Recently, immunotherapy and antibody–drug conjugates have shown great promise in patients resistant to standard therapies [33].

Cancer cells exhibit a buildup of genetic mutations [23], leading to inactivation of the immune system [34], complicating immune surveillance. Immunotherapy, focusing on immune checkpoint therapy, revolutionized the field of cancer treatment by activating immune cells, enabling them to recognize and attack cancer cells. Immune checkpoint inhibitors (ICI) block the checkpoint proteins from binding with their partner proteins, turning the ‘off’ signal to ‘on’, enabling T-cells to destroy cancer cells [34]. These immune checkpoint antagonists led to a paradigm shift in the field of cancer therapy, since ICI antibodies against PD-1 (e.g., nivolumab and pembrolizumab), and against PD-L1 (e.g., atezolizumab, avelumab, and durvalumab) have shown impressive efficacy against many patients with various types of cancer, such as melanoma and NSCLC, with no significant differences between PD-1 and PD-L1 inhibitors. Overall response rates (ORR) depend on the expression of PD-1 and PD-L1 and whether patients are chemo-naïve (ORR up to 48% at high expression) or previously treated patients (11–17% for chemotherapy alone and up to 41% at high expression) [26]. Unfortunately, there is a large population of patients for whom this treatment does not work [23], either because of resistance or due to an imbalance of immune and autoimmune tolerance, which may lead to side effects in patients [34]. A meta-analysis showed that expression of PD-1 and PD-L1 on tumor cells also plays an important role, with low expressors showing no or moderate benefit.

However, recent studies have shown that conventional cytotoxic chemotherapy plays a key role in increasing the efficacy of immunotherapy. Currently, the primary treatment option in, e.g., NSCLC is a combination of chemotherapy with antibodies against PD-1 or PD-L1 receptors [35]. The combination ICI-platinum doublet is usually more effective than either immunotherapy alone or the platinum-doublet [36]. Neo-adjuvant chemoimmunotherapy may lead to an ORR of 53%, but in patients with PD-L1 > 50%, this may increase to 76% [37]. This highlights the essence of understanding how these two therapies affect each other (e.g., how to block immune evasion) and together lead to a better therapeutic outcome.

Several studies have examined CD39 and CD73 expression, chemotherapy-induced modulation and immunotherapeutic targeting, but a comprehensive review of these interconnected findings is lacking. This paper addresses this gap by integrating current evidence on the role of the CD39-CD73-adenosine pathway in chemoimmunotherapy resistance and response, providing a framework for optimizing combination treatment strategies in NSCLC and other high-mutational-burden tumors.

## 2. CD73 and CD39 in Cancer Immunosuppression and Response to Conventional Treatment

### 2.1. Immunosuppressive Functions of the CD39-CD73-Adenosine Pathway

Adenosine counteracts both innate and adaptive immune responses, inhibiting the development and activation of many immune cells, including B cells, neutrophils, NK cells, and macrophages, but mainly affects polarization and activity of T cells, stimulating the polarization of CD4+ T cells towards Treg cells and inhibiting activation and function of effector CD8+ T cells, forming immunosuppressive TME (Table 1). Concurrently, the CD39-CD73 enzymatic cascade reduces the availability of ATP, ADP, and NAD+, agonists for pro-inflammatory P2 purinergic receptors [4,5]. Moesta et al. highlighted the function of CD39 in a variety of immune cell types and how those cells respond to suppression of CD39 [38]. Regulatory T cells (type 1 regulatory T cells), Th17 cells, myeloid-derived suppressor cells (MDSC), and natural killer (NK) cells all have an increased immunosuppressive function when CD39 is present. Inhibition of CD39 results in a reduction in the immunosuppressive activity of regulatory T cells and type 1 regulatory T cells, as well as an improvement in the functional capacity of natural killer (NK) cells and the ability to limit metastatic spread [38,39]. In MDSCs, inhibition of CD39 will likewise have the effect of reversing the suppression of CD8+ T cells. CD39 is a protein that is found on macrophages and is responsible for regulating chemotaxis as well as the balance between inflammatory and regulatory macrophage differentiation. There is an increase in pyroptosis, as well as the release of inflammatory cytokines, when it is suppressed in macrophages [40]. CD39 is responsible for guiding chemotaxis in neutrophils [41], and high levels of CD39 in antigen-presenting cells have a role in reducing inflammation and minimizing pathogenic T-cell responses. An inhibition of CD39 in neutrophils resulted in a reduction in chemotaxis and an increase in the production of IL-8 [42]. On the other hand, inhibition of CD39 in antigen-presenting cells resulted in an increase in maturation and the release of inflammatory cytokines [38,39,43]. Anti-tumor immunity is compromised when there is a high CD73 expression on both tumor cells and immune cells [38,43].

### 2.2. CD73 and CD39 Expression in Cancer Cells

**Figure 2:**
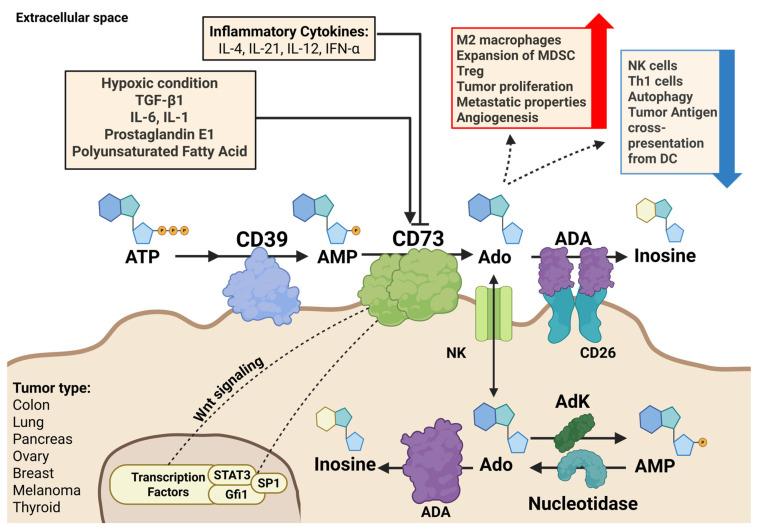
CD73 regulates the tumor microenvironment by converting extracellular AMP into adenosine, whose accumulation promotes immunosuppressive signaling. CD73 expression is regulated at the transcriptional level by factors such as SP1, STAT3, and Gfi1, and may be induced under hypoxic conditions. Additional regulatory inputs include TGF-β1, IL-6, type I interferons, Wnt signaling, cAMP signaling, and exposure to polyunsaturated fatty acids. In contrast, cytokines such as IFN-γ, IL-4, IL-12, and IL-21 have been reported to suppress CD73 expression. Increased CD73 activity and adenosine accumulation are associated with expansion or functional polarization of immunosuppressive cell populations, including M2 macrophages, myeloid-derived suppressor cells (MDSCs), and regulatory T cells (Tregs). These changes contribute to a pro-tumorigenic microenvironment characterized by enhanced tumor proliferation, angiogenesis, and metastatic potential (red arrow). Conversely, elevated adenosine levels impair anti-tumor immune mechanisms. Rather than directly inhibiting all immune cells, adenosine signaling functionally suppresses key effector pathways, including natural killer (NK) cell cytotoxicity, Th1-type responses, autophagy-associated anti-tumor effects, and dendritic cell (DC)-mediated tumor antigen cross-presentation (blue arrow) [53]. Created with BioRender.com.

Although CD73 (ecto-5′-nucleotidase) has been known to play a role in immune dysfunction since the 1970s [45], it was also shown to reduce the efficacy of some antimetabolites in pediatric leukemia, such as 6-mercaptopurine, by degradation of active metabolites [46]. CD73 is highly expressed in subsets of pediatric leukemia [47], and an association was found between CD73 expression and the ex vivo sensitivity of childhood leukemic lymphoblasts to thiopurines [46,48,49]. Mechanistic studies in various preclinical in vitro cellular model systems and (knockout) animal models have provided detailed insight into the function of CD73 and CD39 (Figure 2). Clinical studies focused on the expression of both CD39 and CD73, not only on endothelial cells and various subsets from the immune system, but also on tumor cells. In healthy lung tissue, CD73 and CD39 showed concordant expression patterns in macrophages, bronchial smooth muscle cells, and normal vessels; however, this physiological balance is often disrupted in malignancy [50]. These studies showed that CD73 expression was increased in various cancers, such as melanoma, breast cancer, colon cancer, and lung cancer [51,52]. The mechanism governing CD73 and CD39 regulation, however, is still not completely understood. These clinical studies on the role of CD73 and CD39 in cancer cells usually focused on protein expression. However, few studies evaluated how chemotherapy increases the efficacy of immunotherapy, and what is the role of CD73 and CD39 in this context?

Summarized, in the vast majority of human solid malignancies, CD73 expression is much higher in the tumor compared to its normal counterparts (Table 2) [54]. The enzyme activity and gene expression have been associated with the invasiveness of tumors and the spread of metastatic disease [54]. Extracellular adenosine, produced by CD73, can cause immune evasion, resulting in increased tumor growth and metastasis (Figure 2). In addition to being an immune regulator that is controlled by tumor cells, CD73 is involved in the regulation of various aspects of carcinogenesis [55]. These aspects include proliferation, adhesion/migration, angiogenesis, and metastasis (Table 1). CD73 promotes the expansion of tumor cells by exerting an influence on the cell cycle, apoptosis, and signaling pathways such as EGFR, -catenin/cyclin D1, vascular endothelial growth factor (VEGF), and AKT/ERK. Possibly, by its intrinsic enzymatic activity, CD73 can increase cell-to-cell adhesion, motility, cancer cell invasion, and stemness. Both tumor and host cells need CD73 in order to stimulate tumor angiogenesis. Because of the importance of CD73-A2aR signaling in the process of tumor-associated lymphangiogenesis, it is likely that adenosine blocking drugs minimize the extent of pathological lymphangiogenesis in cancers, which will restrict the spread of tumors [56]. In addition, cancer cell-intrinsic CD73 drives epithelial-to-mesenchymal transition (EMT) by means of the PI3K/AKT and the RICS/Rho GTPase signaling pathway, which will accelerate metastasis [57]. Thus, CD73 expression is commonly associated with a poor prognosis and a poor therapeutic response.

However, there is also evidence for an opposite relation, for which the mechanism is unclear. In some cases, possibly related to some specific tumor types, CD73 expression is associated with a favorable prognosis [55]. In advanced stages of prostate, laryngeal, and high-grade colon carcinomas, CD73 was shown to have a downregulated expression. When compared to normal and well-differentiated early-stage tumors, poorly differentiated and advanced stages of endometrial carcinomas had a lower CD73 expression, while normal and well-differentiated early-stage tumors had a higher CD73 expression. A higher CD73 expression was linked to better overall survival. Actin polymerization was the mechanism via which CD73-generated adenosine preserved epithelial integrity in endometrial malignancies in their early stages [56]. When everything is considered, the function of CD73 in cancer seems to be a confusing one, most likely because CD73 is involved in processes that are not directly related to the promotion of tumor growth [52,62].

### 2.3. Effect of Chemotherapy on CD73 and CD39 in Various Tumor Types

Information on the effect of chemotherapy on the expression of CD73 and CD39 is scattered, both regarding tumor types and drug classes. Therefore, this section summarizes both types of information. In various tumors, CD73 has been linked to the expression of several efflux pumps from the ATP-Binding Cassette (ABC) group, also known as multidrug resistance (MDR) transporters. CD73 expression was associated with doxorubicin resistance in one subtype of breast cancer, triple-negative breast cancer (TNBC) [43]. Doxorubicin is an excellent substrate for several ABC pumps. Doxorubicin therapy led to an elevation of CD73 expression, leading to CD8+ T cell suppression. Other chemotherapeutic drugs, such as carboplatin and paclitaxel, also increased the frequency of CD47+ CD73+ PD-L1+ cells in TNBC cells. CD73 expression was adversely linked with chemotherapeutic sensitivity (Table 3). IL-6 produced from mesenchymal stem/stromal cells increased cisplatin resistance in nasopharyngeal cancer, which was associated with upregulation of CD73 expression. Apparently, with several forms of chemotherapy, CD73 overexpression seemed to counteract the extra ATP generated by dying tumor cells [43].

In glioblastoma multiforme cells, adenosine signaling through A3R and A2bR has been shown to upregulate multidrug resistance protein 1 (MRP1) expression, contributing to resistance against vincristine [68,69]. Moreover, in a mouse glioblastoma model, it was shown that CD73 expression and adenosine signaling via A2bR promote glioblastoma pathogenesis and chemoresistance to temozolomide through increased MRP1 expression.

Carrera-Martínez et al. highlighted the interaction of CD73 and adenosine with adenosine receptors in regulating MRP1 expression in cervical cancer lines [70]. A2aR activation by adenosine seemed to increase MRP1 expression. Inhibition of CD73 or A2aR—using siRNA and the selective antagonist ZM241385, respectively—resulted in decreased MRP1 expression and enhanced sensitivity of cervical cancer cells to cisplatin. Since this effect was stronger than that observed with MK-571, a direct MRP1 inhibitor, this suggests that targeting CD73 or A2aR modulates other resistance mechanisms to cisplatin, since platinum analogs are not a substrate for any ABC-transporter [71]. Notably, CD73 downregulation or A2aR blockade significantly increased the sensitivity of cervical cancer cells to cisplatin, as shown by a reduction in the concentration needed to affect the growth of cervical cancer cells down to 25–39% or 29–64%, respectively. These findings suggest that inhibiting the CD73/ADO/A2aR axis may be a promising strategy to overcome chemoresistance in cervical cancer [72].

Ziebart et al. investigated the influence of chemotherapy on adenosine-producing B cells in patients with squamous cell carcinoma [73]. CD39’s enzymatic activity effectively hydrolyzes ATP to AMP. Pre-Cultured B cells were isolated and treated with one cytostatic drug to quantify the activity of CD39 and determine the impact of chemotherapy. B cells treated with 2 µg/mL (6.6 µM) cisplatin (*p* < 0.05) or 5 µg/mL (5.9 µM) paclitaxel (*p* < 0.01) showed a reduced ATP hydrolysis compared to non-treated control B cells. Furthermore, even at lower 1–3 µg/mL concentrations, paclitaxel reduced ATP consumption significantly. In contrast, methotrexate (MTX) induced a higher rate of ATP hydrolysis. For all samples, there was a substantial association between ATP use and CD39 expression (*p* < 0.0001), showing a direct link.

It can be concluded that most conventional chemotherapeutics, including those used for the treatment of NSCLC, increase CD73 and CD39 expression (Table 4), leading to resistance. However, the molecular interactions leading to this effect remain unclear, also because the effects were different between therapeutics. However, since the most common effect was an increase, it was hypothesized that targeting CD39 and/or CD73 might increase the anticancer effect of these drugs.

## 3. Targeting the CD39/CD73 Pathway in Treatment

Many compounds that affect adenosine metabolism, specifically targeting CD73 and/or CD39, have recently been reviewed [5,75,76]. Several groups developed potent CD73 and CD39 inhibitors, but their evaluation was usually limited to characterization of inhibition, without further detailed analysis. Several compounds are discussed below [77,78].

### 3.1. IPH5201 and IPH5301 Antibodies Block the CD39/CD73 Pathway in Model Systems

The use of ICIs has significantly improved therapeutic interventions in cancer. Some cancer patients respond well to immune checkpoint blockade (ICB) treatment, but many patients either do not react or develop resistance after an initial response. Several forms of cancer are not sensitive to ICIs. This might be related to the fact that the TME comprises alternative immunosuppressive pathways as well as immunosuppressive pathways that are triggered by medication. It is essential to find new inhibitory signals to stop tumor growth and evasion. CD73 and CD39 seem to play a crucial role in ICB treatment that is administered via ICI.

Initially, antibodies were investigated for their potential therapeutic efficacy. A therapeutic synergy was found when A2aR and ICB were both targeted at the same time [79]. For instance, the levels of CD73 increased in melanoma patients who received anti-PD-1 medication. In addition, comprehensive immunological profiling indicated the presence of a macrophage population that exhibited high levels of CD73 expression. In a mouse model of glioblastoma, the absence of CD73 led to an improvement in the efficacy of treatment with anti-PD-1 and anti-CTLA-4 antibodies [80].

**Figure 3:**
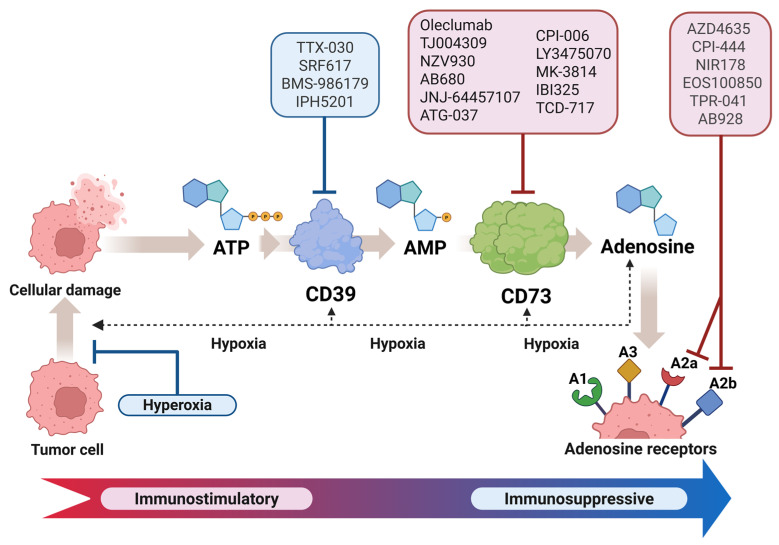
An immunosuppressive microenvironment is created by extracellular adenosine. Both tumor cells and enzymes dephosphorylate ATP, resulting in the release of adenosine into the bloodstream. Immune cells are activated when they detect ATP, but adenosine is an anti-inflammatory molecule that signals primarily via the A2b and A2a receptors on a broad spectrum of innate and adaptive cells [81]. Drugs in clinical trials are shown in red, while those that target hypoxia and CD39 are shown in blue; both are in preclinical research. Based and adapted from [78]. Created with BioRender.com.

Adenosine is produced by the successive action of CD39 and CD73 ecto-enzymes, which is one of the factors that lead to the development of an immunosuppressive tumor microenvironment. Several antibodies (Figure 3) were developed to target CD39 and CD73, and to efficiently block the adenosine pathway. The rationale for this was to avoid immunogenic ATP from being hydrolyzed into immunosuppressive adenosine. These antibodies restore T cell activation, which in turn stimulates antitumor immunity. Additionally, dendritic cells and macrophages were also activated in the process. The anti-tumor activity of the ATP-inducing anticancer drug oxaliplatin was boosted by the administration of IPH5201 in a human CD39 knock-in mouse model. These results provide a rationale for the use of anti-CD39 and anti-CD73 monoclonal antibodies in the treatment of cancer, particularly in conjunction with immune checkpoint inhibitors and chemotherapeutic agents [65].

### 3.2. Silencing Tumor-Intrinsic CD73 in Model Systems

Baghbani et al. [82] evaluated cisplatin treatment and transfection with CD73-small interfering RNA (siRNA) on the survival of A549 and NCI-H1299 NSCLC cells. A decrease in cell survival and an increase in chemosensitivity to cisplatin were seen after the transfection of CD73-siRNA into A549 and NCI-H1299 cells. The use of CD73-siRNA in NSCLC cells resulted in a considerable increase in apoptosis, a pause in the cell cycle, and a reduction in tumor migration. Additionally, when cisplatin was combined with CD73-siRNA transfection, the anti-tumor effects of cisplatin against NSCLC cells were significantly increased. When cisplatin treatment is combined with CD73-siRNA transfection, NSCLC may be considerably more responsive to chemotherapy by boosting the anti-tumor effects of cisplatin on NSCLC [82].

### 3.3. Clinical Trials with Compounds Inhibiting CD73 and CD39

Table 5 summarizes clinical trials collected from clinicaltrials.gov, both completed and ongoing. The number of patients for each compound is still limited in ongoing studies, so it is not clear yet how many patients might benefit from each treatment. There are more clinical trials involving compounds inhibiting CD73 (Oleclumab, S095024, Mavrostobart, Uliledlimab, Mupadolimab, LY3475070, NZV930) compared to drugs affecting CD39 (TTX-030, SRF617). CD73 inhibitors are often combined with immune checkpoint inhibitors (such as Durvalumab, Pembrolizumab) or chemotherapy to improve immune responses and antitumor activity, whereas Durvalumab is also used in interventions in combination with other compounds. Clinical trials with Oleclumab as monotherapy demonstrated its tolerable safety profile but no significant antitumor effect [83], indicating that Oleclumab needs to be combined with other compounds for improved efficacy, similar to Durvalumab. In stage III NSCLC (NCT03822351), oleclumab plus durvalumab improved overall response rate (ORR) and prolonged progression-free survival (PFS) compared with durvalumab alone [84]. In NSCLC (NCT03794544), the combination achieved a 19% major pathological response (MPR) [85]. In EGFR-mutant NSCLC (NCT02503774), oleclumab ± durvalumab showed pharmacodynamic activity and manageable safety, with evidence of antitumor activity even in tumors typically resistant to immunotherapy [85]. Conversely, in advanced prostate cancer (NCT02740985/NCT04089553), combinations including oleclumab demonstrated minimal antitumor activity despite acceptable tolerability. Other CD73-targeting agents have shown encouraging safety profiles. LY3475070 combined with pembrolizumab was generally well tolerated, with fatigue, diarrhea, and rash as the most common treatment-related adverse events (TRAEs), and grade 3–4 TRAEs occurring in a minority of patients. Oleclumab plus osimertinib demonstrated moderate activity with acceptable tolerability in previously treated EGFR-mutated NSCLC (NCT03381274) [86]. NZV930 [87] and mupadolimab have also been evaluated alone or in combination with PD-1/PD-L1 or A2aR inhibitors, with acceptable safety in early-phase studies.

Among CD39-targeting therapies, TTX-030 combined with budigalimab and FOLFOX showed a 61% response rate as first-line treatment in advanced gastric/GEJ cancer, with manageable safety across PD-L1 combined positive score subgroups [94]. SRF617 demonstrated good tolerability, with no dose-limiting toxicities reported; the most common adverse events varied by cohort and included fatigue, nausea, constipation, pruritus, anemia, and neutropenia.

Overall, the combination of CD73 inhibitors with ICIs such as durvalumab or pembrolizumab has demonstrated encouraging clinical activity, including increased response rates and signals of improved survival outcomes in selected settings [84,93]. Safety profiles across studies are generally manageable, with predominantly low-grade adverse events and relatively infrequent severe toxicities. Nevertheless, the limited size and early-phase design of most trials necessitate further randomized studies to confirm efficacy, better define survival benefit, and optimize safety. Oleclumab, both as a single agent and in combination with anti-PD-1/PD-L1 antibodies and chemotherapy, has been demonstrated to suppress tumor growth in multiple models by enhancing anti-tumor immune responses [93]. However, the withdrawal of some studies (NCT04262375, NCT04262388) additionally highlights the biological and clinical challenges associated with targeting the adenosine pathway and underscores the need for improved patient selection and combination strategies.

## 4. EGFR-Mutated and KRAS-Mutated Non-Small Cell Lung Cancer

Many clinical trials with CD39 and/or CD73 inhibitors focus on combinations with conventional chemotherapy, raising the question whether these compounds might also play a role in tyrosine kinase-directed therapy, such as EGFR-TKI, erlotinib and gefitinib. Approximately 10–15% of NSCLC patients have EGFR activating mutations (EGFR Exon 19 Deletion, Exon 21 L858R) [95] and used to be treated with a first-generation EGFR-TKI, erlotinib or gefitinib. It is argued that because of the low immunogenicity and immunological ignorance, immune checkpoint inhibitor treatment would be ineffective in these EGFR-mutant NSCLC. Regardless of the initial response, acquired resistance after 9–18 months seems to be unavoidable. As a result, the development of innovative therapeutic techniques is more vital than ever. However, CD39 and CD73 ecto-nucleotidases may play a role in immunosuppressive effects such as immune metabolic dysfunction of EGFR mutations, characterized by the antigen production and release of extracellular adenosine [54,56].

When signaling pathways, notably in the mutated KRAS pathway, are active, CD73 protein expression will increase, and given its active state, will affect the immune system, rendering PD-1 and PDL-1 more active. These patients are also resistant to the TKIs used to treat EGFR-activating mutations [57], and CD73 inhibitors may be beneficial.

## 5. Perspectives

Even though treatments for NSCLC have improved a lot in recent years, patients with most forms of lung cancer still have a poor prognosis. Immunotherapy has been shown to restore the immune system’s ability to attack cancer cells, although tumors and immune cells may become resistant to ICIs. CD73 is an excellent therapeutic target for cancer treatment for various reasons: (1) high CD73 expression by cancer cells and host cells, such as immune cells, creates an adenosine-rich TME that suppresses the immune system; (2), CD73 is a protein intrinsic for cancer cells and supports their growth; (3) the enzyme activity of CD73 stimulates tumors to grow and spread. Since many therapies seem to change the expression of CD73 and its function, CD73 is an attractive target, along with other therapeutic agents.

The precise role of CD73 and adenosine signaling in the efficacy of ICI on both tumor cells and invading immune cells is unknown. Targeting CD73/CD39 and adenosine signaling may bypass initial resistance to immunotherapy. However, further preclinical and clinical studies are necessary to elucidate the role of adenosine. These studies should give insight into how to translate adenosine signaling inhibition to target the TME’s abnormal immune-metabolism regulation. Such research should also focus on the effect of conventional chemotherapy on ATP homeostasis and the role of CD73 and CD39. Treatment with several forms of chemotherapy will induce CD73 expression concurrently with the generation of extra ATP by dying tumor cells. Treatment of NSCLC cancer cells with CD73-siRNA transfection increased the cytotoxicity of cisplatin treatment. More evidence for the crucial role of both CD73 and CD39 in ICI treatment includes the potentiating effect of both anti-CD39 and anti-CD73 monoclonal antibodies in the experimental treatment of cancer, particularly in conjunction with ICIs and chemotherapeutic agents.

CD73 should also be considered as a possible biomarker for prognosis and/or prediction, and a better understanding of how CD73 blockade and combination therapy work is mandatory. Adenosine signaling is important in carcinogenesis, and multiple studies have shown that overexpression of CD73 in cancer cells is associated with a poor outcome in NSCLC patients. Therefore, it is necessary to get a better understanding of the molecular pathways that contribute to tumor development and immune evasion. Next, the function of the enzymes downstream of CD73, ADA, and PNP in the adenosinergic effect in tumors and TME should be investigated in more detail. A high activity of these enzymes leads to a rapid degradation of adenosine, and it should be clarified how this affects both adenosine and CD73-mediated downstream signaling.

## 6. Discrepancy

Despite numerous studies investigating the role of adenosine in cancer progression, the exact prognostic significance of CD39 and CD73 expression in NSCLC remains unclear due to several interconnected factors. Most importantly, CD39 and CD73 are expressed at different levels across multiple cellular compartments within the tumor microenvironment, including cancer cells, cancer-associated fibroblasts, tumor-infiltrating lymphocytes, and myeloid-derived suppressor cells. The spatial localization of these ectonucleotidases significantly influences prognosis: high CD39 and CD73 expression in tumor stroma correlates with poor recurrence-free survival at 5 years, whereas CD39+ CD103+ CD8+ T cells located in the tumor nest predict improved outcomes [50,96]. This compartment-specific prognostic impact explains why studies measuring total tissue expression without spatial resolution may yield contradictory results. Additionally, the tumor microenvironment composition varies substantially across different cancer types and individual patients, influenced by factors such as hypoxia, which can affect CD39 and CD73 expression patterns [50]. Expression patterns and prognostic significance differ substantially between adenocarcinoma and squamous cell carcinoma. CD73 expression is significantly higher in adenocarcinoma than in squamous cell carcinoma and is particularly elevated in tumors harboring EGFR or KRAS mutations [54,58]. This molecular heterogeneity means that studies with varying proportions of adenocarcinoma versus squamous cell carcinoma, or different frequencies of oncogenic drivers, will report divergent prognostic associations. The literature reveals opposing prognostic effects depending on study design and patient populations. Inoue et al. found that high CD73 expression in tumor cells independently predicted worse overall survival and recurrence-free survival in a predominantly adenocarcinoma cohort [58]. Conversely, Koppensteiner et al. demonstrated that in tumors with similar adenocarcinoma and squamous cell carcinoma representation, high CD39 expression in tumor cells combined with low CD73 correlated with better overall survival, while high stromal expression of both markers predicted poor outcomes [96]. Additionally, Shao et al. reported that low CD39 expression in lung adenocarcinoma was associated with poor prognosis and reduced immune infiltration [97]. These contradictions likely reflect differences in the cellular source of expression (tumor vs. stroma vs. immune cells), histological composition, and mutational landscape.

## 7. Conclusions

CD39 and CD73 contribute to tumor progression through metastasis, angiogenesis, and immune modulation. Inhibitors targeting these ectonucleotidases show promise as cancer therapeutics, either alone or combined with chemotherapy and immunotherapy. However, their widespread expression in normal tissues necessitates careful monitoring for adverse effects. Ongoing clinical trials will clarify the therapeutic efficacy and safety profile of CD73 and CD39 inhibition, with pre-treatment and serial measurement of CD73, CD39, PD-1, and PD-L1 expression recommended to optimize patient selection and treatment monitoring.

## Figures and Tables

**Table 1:** Summary of CD73-generated adenosine effect in preclinical studies on various cell types present in the tumor microenvironment.

Cell Type	Model of the Study	Description	Ref.
T cells	Preclinical data: in vitro T-cell assays; murine tumor models	Inhibit the proliferation, differentiation, activation, cytotoxicity of T cells, and cytokine release.	[38]
Treg cells	Preclinical data: in vitro Treg suppression assays; murine tumor models	Promote proliferation/differentiation, CTLA-4/PD-1 expression, suppressive activity, tolerance, and anergy.	[38,41]
B cells	Patient-derived and preclinical data: B cells isolated from cancer patients; in vitro co-culture assays	Inhibit tumor infiltration, IgG2b production, and IL-17/IFN-γ by CD4+ T cells CD73 expression promotes regulatory B cells (Breg cells) with immunosuppressive activity towards T cells	[39,44]
NK cells	Preclinical data: in vitro NK-cell functional assays; murine tumor models	Inhibit activation, maturation, cytotoxicity of NK cells, and cytokine release.	[38]
DCs	Preclinical data: in vitro-differentiated DCs; murine tumor models	Inhibit activation, maturation, and antigen presentation ability	[38]
MDSC	Preclinical and translational data: murine tumor models; analysis of tumor-infiltrating myeloid cells	Promote tumor infiltration, differentiation, and suppressive activity	[43]
TAMs	Preclinical data: murine tumor models; in vitro macrophage polarization assays	Promote the switch from M1 to M2 phenotype and increase suppressive activity.	[38]
CAFs	Preclinical data: stromal–tumor cell co-culture systems; murine tumor models	Promote non-redundant A2bR-CD73 circuit and tumor-promoting activity	[35,37,40]
Tumor cells	Preclinical and translational data: tumor cell lines; murine tumor models; human tumor sample analyses	Promote proliferation, angiogenesis, invasion/migration, and metastasis	[43]

Abbreviations: A2bR—adenosine A2a receptor; Breg—regulatory B cells; CAF—cancer-associated fibroblasts; CTLA-4—cytotoxic T-lymphocyte-associated protein 4; DCs—dendritic cells; IFN-γ—interferon gamma; IgG2b—immunoglobulin G subclass 2b; IL-17—interleukin 17; M1—M1 macrophages; M2—M2 macrophages; MDSC—myeloid-derived suppressor cells; NK—natural killer; PD-1—programmed cell death protein 1; TAMs—tumor-associated macrophages; Treg—regulatory T cells.

**Table 2:** Summary of the protein expression of CD73/CD39 in malignant cells.

Tumor Type	Expression		# of Samples	Ref.
	CD73	CD39		
NSCLC	Neg. in 55.1%. Low/High pos. in 16.3% and medium in 12.3%	Neg. in 76.5%, low expression in 23.5%	98	[50]
NSCLC	Higher in adenocarcinomas, in women and non-smokers		653	[58]
Endometrial cancer	Present in stromal and epithelial structures of tumor	Present in the stroma; 2-fold higher in serous endometrial adenocarcinoma than in endometrioid	29	[59]
Bladder Cancer		Males: neg. 30%, low 26%, high 44%; Females: neg. 21%, low 33%, high 46%; NMI: neg. 11%, low 36%, high 52%; MI: neg. 53%, low 15%, high 32%	162	[60]
Uveal melanoma	Higher in patients who did not receive adjuvant proton therapy		12	[61]
	No significant associations were found between CD39/CD73 expression and clinical parameters			

Abbreviations: NSCLC—Non-Small Cell Lung Cancer; NMI—Non-Muscle Invasive (bladder cancer); MI—Muscle Invasive (bladder cancer); Neg.—negative expression; Pos.—positive expression.

**Table 3:** Clinical/biological effects of low & high CD73/CD39.

Tumor Type	Model	CD73/CD39 Expression	Clinical/ Biological Outcome	Key Finding	Ref.
Endometrial Ovarian	Human tissues, mice tissues, cell lines	CD73-low (tumor epithelium)	↑ tumor growth	Loss of CD73 disrupts epithelial barrier integrity, promoting tumor progression	[63]
Pancreatic	Human tissues	CD39-high (tumor tissue)	↑ survival	High CD39 mRNA post-resection associated with improved long-term survival	[64]
Melanoma, CRC, Fibrosarcoma, TNBC, Ovarian, Lymphoma	Murine tissues, cell lines	CD73/CD39-high (tumor site)	↑ metastasis	Dual overexpression creates immunosuppressive TME that inhibits anti-tumor immunity	[65]
Breast	Murine tissues, cell lines	CD73-high (tumor cells)	↑ progression	CD73 upregulation linked to oncogenic pathways (WNT, EMT, TP53/KRAS mutations, TGF-β)	[66]
Renal Cell Carcinoma	Human tissues, The Cancer Genome Atlas	CD73-high (tumor tissue)	↓ survival	CD73 overexpression predicts poor prognosis; promotes immunosuppression and angiogenesis	[67]

↑—increased, ↓—decreased.

**Table 4:** The effect of conventional chemotherapy on CD73/CD39 expression in preclinical settings.

Protein	Conventional Drug	Effect	Ref.
CD73	Doxorubicin, carboplatin, gemcitabine, paclitaxel	Increased expression in platinum-resistant ovarian cancer. IL-6-mediated cisplatin resistance in nasopharyngeal cancer	[43]
CD73	Doxorubicin	Resistance in mouse BC cells with CD73 overexpression, mediated by A2a adenosine receptor activation	[52]
CD39	Methotrexate	Increased expression	[73]
CD39	Platinum-based agents	Decrease expression	[73]
CD39	Cytarabine, anthracyclines	Increased expression	[74]

**Table 5:** Clinical trials of antibodies and other compounds targeting the adenosine pathway.

Clinical Trial (Reference)	Type of Tumor	Target (Protein/Drug)	Combined with	Number of Patients	Phase	Status
NCT02740985 [88]	Advanced solid malignancies, NSCLC, CRC	CD73 (Oleclumab)	AZD4635 (A2aR antagonist) + Durvalumab (anti-PD-L1 antibody)	313	1	Completed
NCT03822351 [84]	Stage III NSCLC	CD73 (Oleclumab)	Durvalumab (anti-PD-L1 antibody)	189	2	Active, not recruiting
NCT03794544 [85]	NSCLC	CD73 (Oleclumab)	Durvalumab (anti-PD-L1 antibody)	21	2	Completed
NCT06162572 [89]	NSCLC	CD73 (S095024, Sym024)	Cemiplimab (anti-PD1 antibody)	176	1b/2	Recruiting
NCT05431270	NSCLC, PDAC	CD73 (Mavrostobart)	Gemcitabine + nab-Paclitaxel; Docetaxel; Pemetrexed; Gemcitabine; Carboplatin + Pemetrexed; Pembrolizumab (anti-PD1 antibody) + Carboplatin + Pemetrexed. Tislelizumab (anti-PD1 antibody)	40	1/2	Recruiting
NCT03801902 [90]	NSCLC	CD73 (Oleclumab)	Durvalumab + Standard Radiation Therapy	48	1	Completed
NCT04262375	NSCLC, RCC	CD73 (Oleclumab)	Durvalumab		2	Withdrawn
NCT03381274 [86]	NSCLC	CD73 (Oleclumab)	Osimertinib (TKIs) or AZD4635 (A2aR antagonist)	43	1b/2	Active, not recruiting
NCT04262388	NSCLC, PDAC, HNSCC	CD73 (Oleclumab)	Durvalumab		2	Withdrawn
NCT05001347	Gastrointestinal cancer, head and neck cancer, NSCLC, OC, TNBC	CD73 (Uliledlimab)	Atezolizumab (anti-PD-L-1)	25	2	Active, not recruiting
NCT03454451 [91]	Bladder cancer, cervical cancer, colorectal cancer, endometrial cancer, mCRPC, NSCLC, non-Hodgkin lymphoma, OC, pancreatic cancer, RCC, sarcoma, HNSCC, TNBC	CD73 (Mupadolimab)	Mupadolimab alone or combined with: ciforadenant (A2aR antagonist); or pembrolizumab (anti-PD1 antibody)	117	1/1b	Active, not recruiting
NCT04148937	Advanced cancers: breast cancer, pancreatic cancer, lung cancer, kidney cancer, melanoma, prostate cancer, OC	CD73 (LY3475070)	Pembrolizumab (anti-PD-1)	52		Completed
NCT04672434 [92]	HNSCC, NSCLC, PDAC, cholangiocarcinoma, colorectal cancer, gastric cancer, esophageal cancer, mesothelioma, cervical cancer	CD73 (S095024/Sym024)	Sym021 (anti-PD-1 antibody)	48	1	Recruiting
NCT03549000 [87]	Colorectal cancer, mCRPC, NSCLC, OC, PDAC, RCC, TNBC	CD73 (NZV930)	NZV930 alone or combined with spartalizumab (anti-PD-L1 antibody) or NIR178 (antagonist of the A2aR)	127	1/1b	Completed
NCT02503774 [93]	EGFR-mutant NSCLC	CD73 (Oleclumab)	Durvalumab (anti-PD-L1 antibody)	192	1	Completed
NCT04306900	Gastroesophageal cancer, colorectal cancer, NSCLC, urothelial cell cancer, pancreatic cancer, bladder cancer	CD39 (TTX-030)	Budigalimab (anti-PD-1 antibody) or FOLFOX6 or docetaxel or nab-paclitaxel or gemcitabine or pembrolizumab (anti-PD-1 antibody)	185	1/1b	Active, not recruiting
NCT04336098	Pancreatic cancer, gastric cancer, GEJ adenocarcinoma, NSCLC	CD39 (SRF617)	gemcitabine, albumin-bound paclitaxel, pembrolizumab (anti-PD-1 antibody)	85	1	Active, not recruiting

Abbreviations: CRC—colorectal cancer; GEJ—gastroesophageal junction; HNSCC—head and neck squamous cell carcinoma; mCRPC—metastatic castration-resistant prostate cancer; NSCLC—non-small cell lung cancer; OC—ovarian cancer; PADC—pancreatic ductal adenocarcinoma; PD-1—programmed death-1 receptor; PD-L1—programmed death ligand-1; RCC- renal cell carcinoma; TKIs—small-molecule tyrosine kinase inhibitors.

## Data Availability

The original contributions presented in this study are included in the article. Further inquiries can be directed to the corresponding author.

## Abbreviations

The following abbreviations are used in this manuscript:

A2aR	adenosine A2a receptor
A2bR	adenosine A2b receptor
Ado	adenosine
AdK	adenosine kinase
AMP	adenosine monophosphate
ADP	adenosine diphosphate
ATP	adenosine triphosphate
CRC	colorectal cancer
EGFR	epidermal growth factor receptor
ENTs	equilibrative nucleoside transporters
GEJ	gastroesophageal junction
HNSCC	head and neck squamous cell carcinoma
ICI	immune checkpoint inhibitor
MDR	multidrug resistance
MDSC	myeloid-derived suppressor cells
MHC-I	major histocompatibility class I
MI	muscle invasive
NMI	Non-muscle invasive
NK	natural killer
NSCLC	non-small cell lung cancer
OC	ovarian cancer
PADC	pancreatic ductal adenocarcinoma
PD-1	programmed cell death protein 1
PD-L1	programmed death ligand 1
PNP	purine nucleoside phosphorylase
Pos.	positive expression
RCC	renal cell carcinoma
SCID	severe combined immunodeficiency disease
SCLC	small-cell lung cancer
TKI	tyrosine kinase inhibitor
TNBC	triple negative breast cancer
VEGF	vascular endothelial growth factor
Neg.	negative expression
